# Supplementary material for: Constant strain accumulation rate between major earthquakes on the North Anatolian Fault
Source: Nat Commun. 2018 Apr 11;9:1392. doi: 10.1038/s41467-018-03739-2 (PMC5895838; doi:10.1038/s41467-018-03739-2)
Supplement: Supplementary file 1 — Supplementary Information [file 41467_2018_3739_MOESM1_ESM.pdf]

Supplementary Information for Hussain et al. “**Constant strain accumulation rate between major earthquakes on the North Anatolian Fault**”

Contents of this file

1. Table 1 and Table 2
2. Figures 1 to 10

Supplementary Table 1: Data coverage for each Envisat track used in this study.

Track	Geometry	Time span	No. of images	Ifgs created	Ifgs used	ERA-I improvement[a], %
64	Descending	20040103 - 20100327	21	61	27	13
293	Descending	20040119 - 20101004	20	46	28	3
21	Descending	20021106 - 20090930	25	76	35	8
250	Descending	20031212 - 20100723	38	115	59	1
479	Descending	20031228 - 20100704	30	90	50	12
207	Descending	20040113 - 20100928	40	88	53	10
436	Descending	20030703 - 20100318	36	96	65	2
164	Descending	20031206 - 20100717	44	104	75	1
393	Descending	20031013 - 20100104	29	70	59	-6
350	Descending	20030207 - 20100730	34	79	41	4
429	Ascending	20021204 - 20090819	21	63	29	4
157	Ascending	20030404 - 20080328	19	44	26	7
386	Ascending	20040229 - 20080727	12	29	17	-2
343	Ascending	20040610 - 20100415	14	27	20	6
71	Ascending	20040103 - 20090829	19	48	29	16
28	Ascending	20040728 - 20100707	14	30	21	10
128	Ascending	20041013 - 20100609	12	25	17	-3

[a] The percentage noise reduction for each track after the ERA-I weather model correction for tropospheric noise. We remove a ramp from each interferogram before computing the standard deviation.

Supplementary Table 2: θ_{rot} values used for the rotation correction (see Methods).

Profile ID[a]	Lon. extent (°E)	θ_{rot} (mm/yr/km)
a	29 - 31.25	0.0255
b	31.25 - 33.5	0.0307
c	33.5 - 35.5	0.0138
d	35.5 - 38	0.0213
e	38 - 40	0.0308

[a] GNSS profiles shown in Fig 3b and Supplementary Fig. S7.

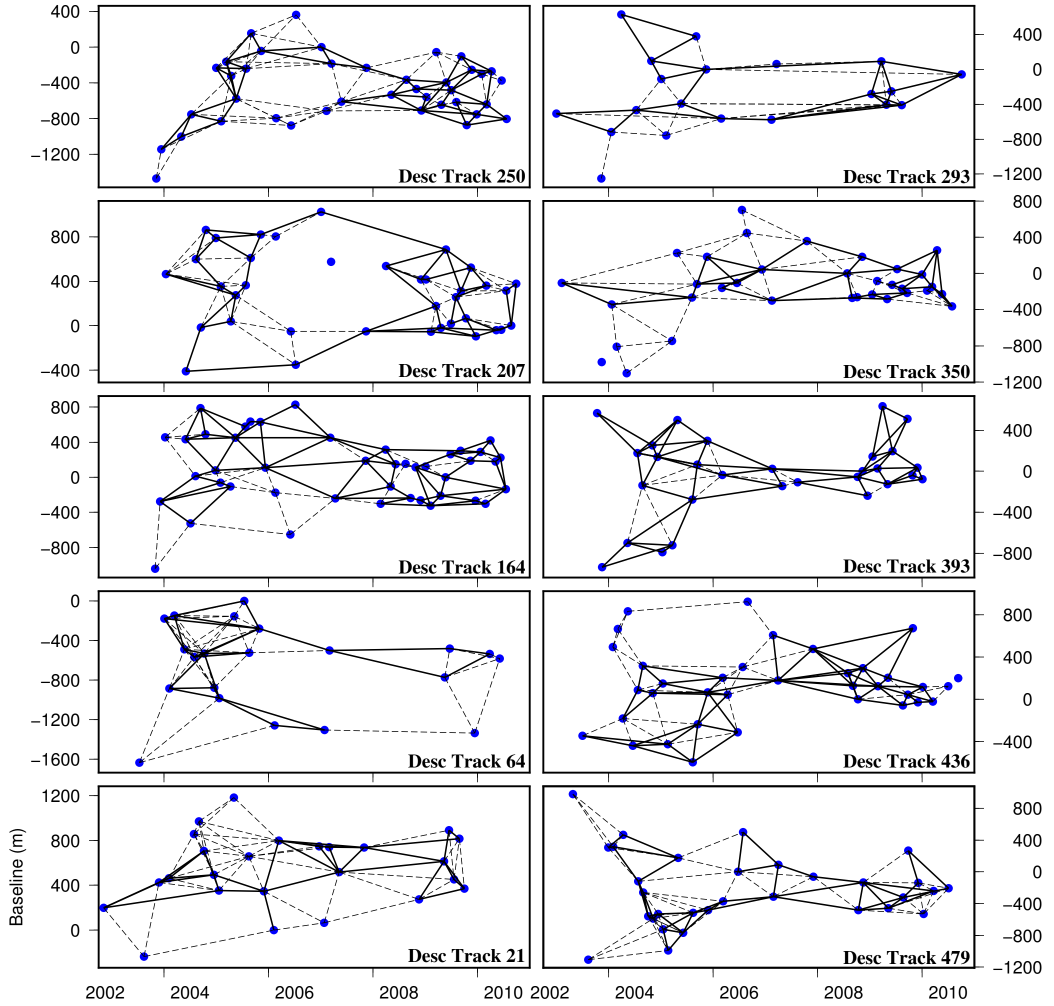

Supplementary Figure 1: Plot showing the timing and relative perpendicular baseline of each acquisition's orbit for each of the descending tracks processed in this study. The combination of dashed and solid lines represent all the interferograms created. The solid black lines are the interferograms used to estimate the average line-of-sight velocity over the time period.

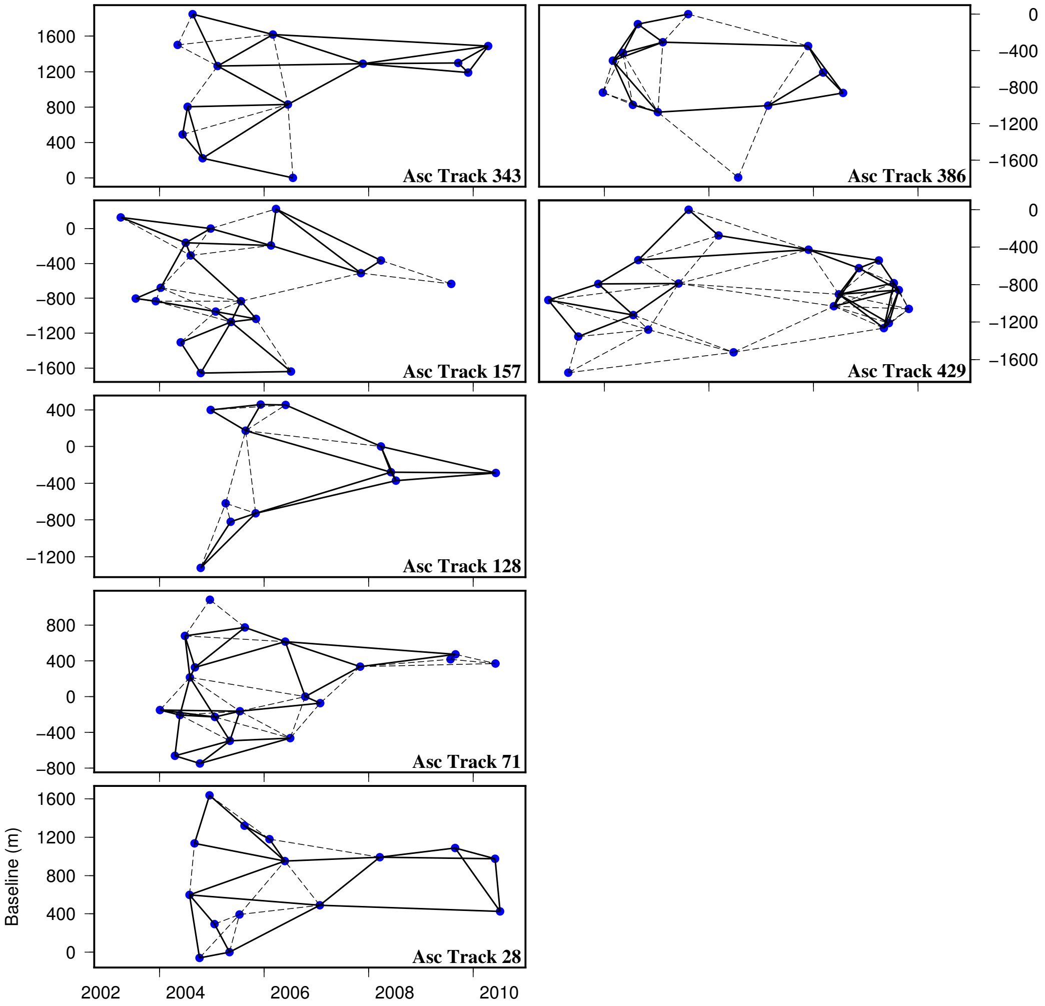

Supplementary Figure 2: Perpendicular baseline vs time plots for each of the ascending tracks processed in this study.

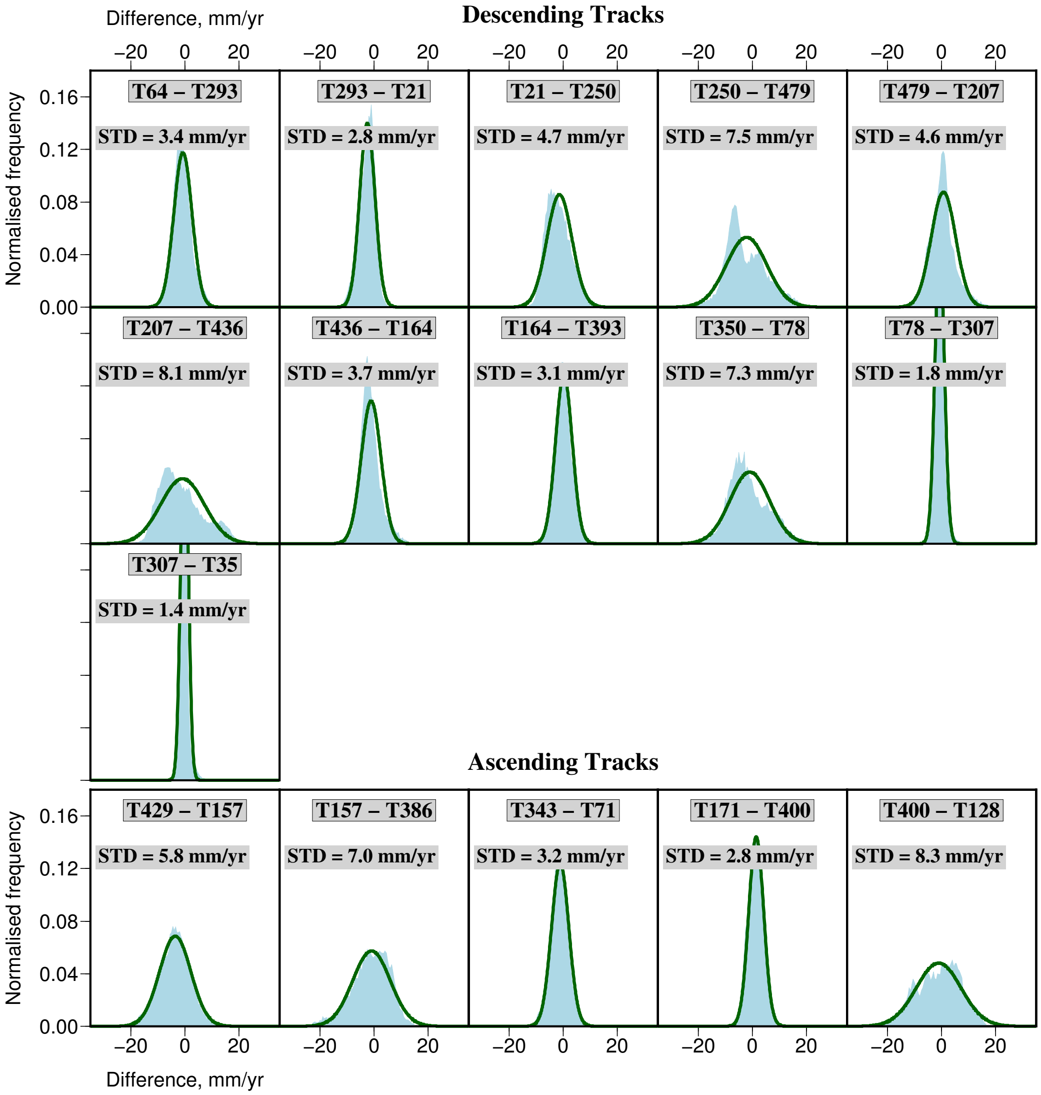

Supplementary Figure 3: Histograms of the differences between estimates of the horizontal component of the LOS velocity at pixels in the regions of overlap between neighbouring tracks. The green line on each histogram is the best fitting Gaussian, and the number above is the standard deviation of this Gaussian.

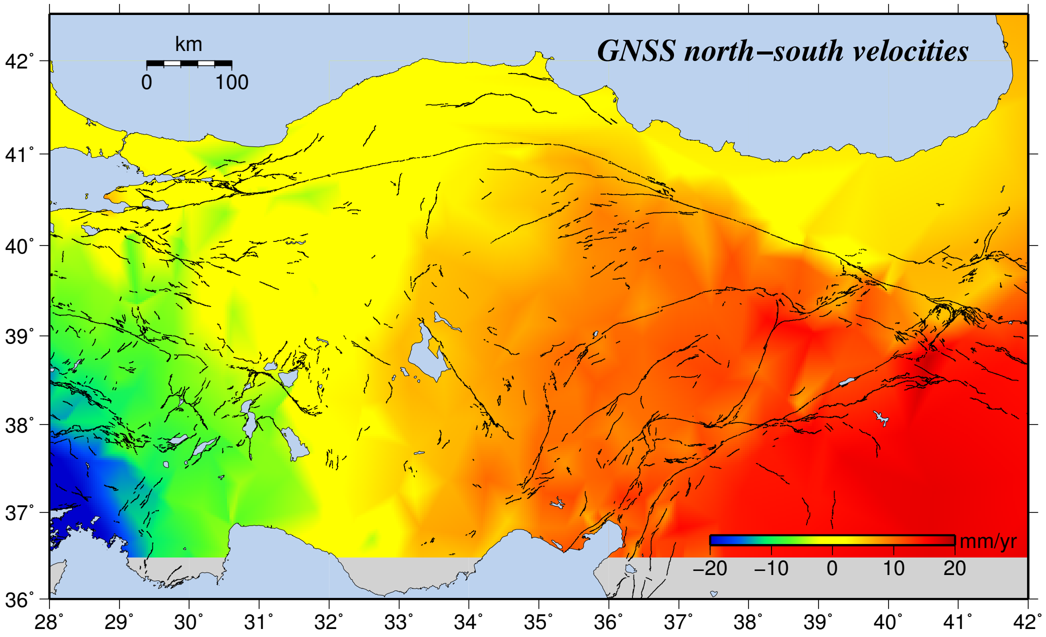

Supplementary Figure 4: Interpolated GNSS north component velocities from the Global Strain Rate Model. Positive velocities indicate motion to the north while negative velocities implies motion to the south relative to Eurasia.

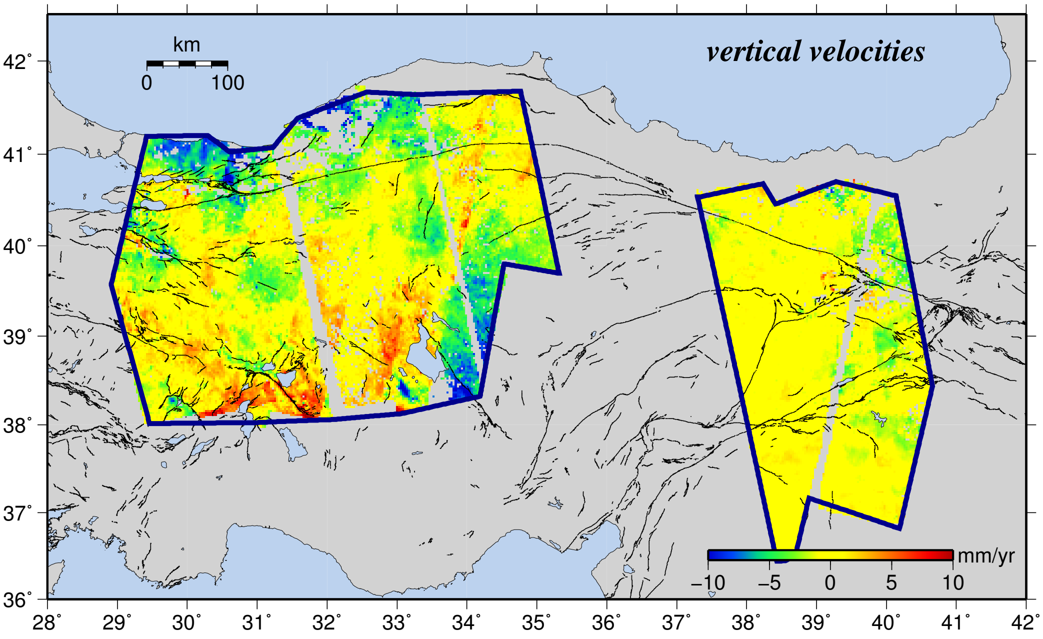

Supplementary Figure 5: Vertical velocity component (mm yr^{-1}) relative to the mean, calculated for the regions with both ascending and descending information.

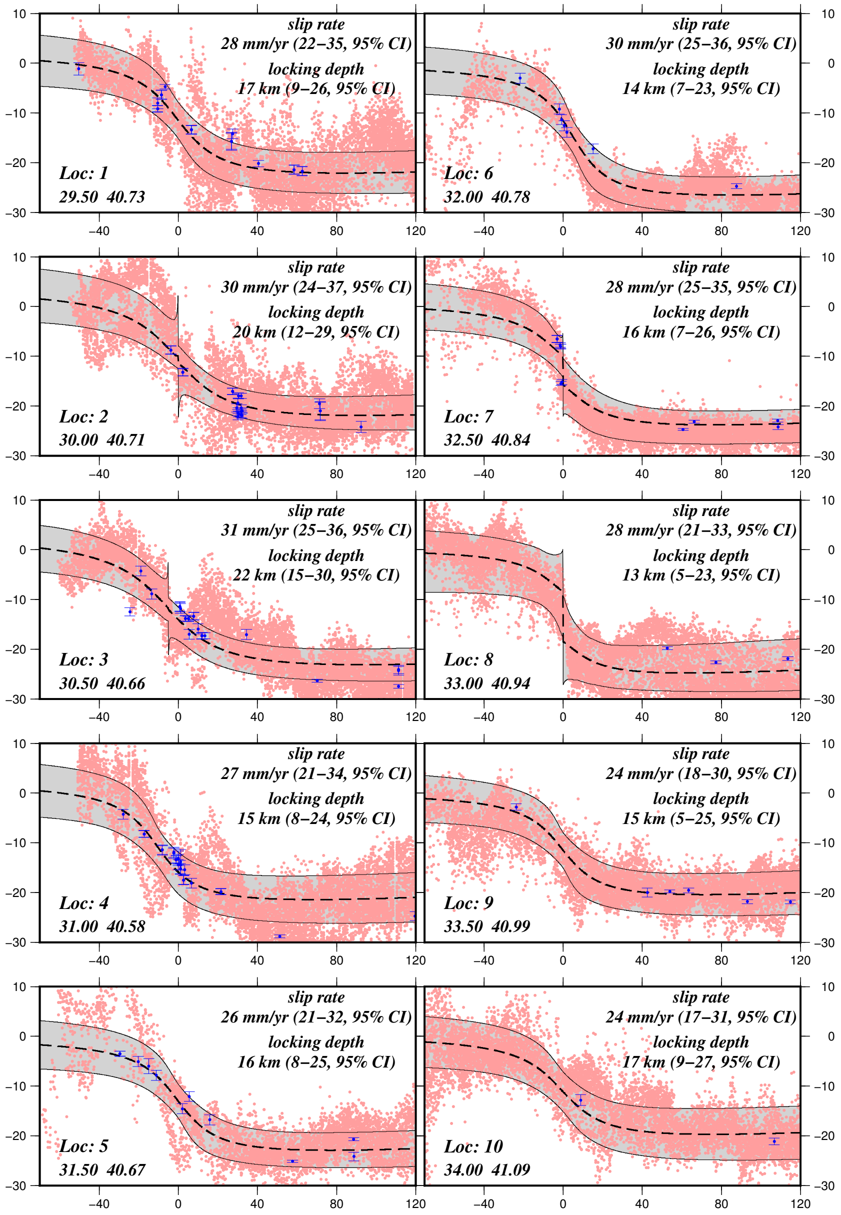
slip rate
28 mm/yr (22–35, 95% CI)
locking depth
17 km (9–26, 95% CI)
Loc: 1
29.50 40.73
slip rate
30 mm/yr (25–36, 95% CI)
locking depth
14 km (7–23, 95% CI)
Loc: 6
32.00 40.78
slip rate
30 mm/yr (24–37, 95% CI)
locking depth
20 km (12–29, 95% CI)
Loc: 2
30.00 40.71
slip rate
28 mm/yr (25–35, 95% CI)
locking depth
16 km (7–26, 95% CI)
Loc: 7
32.50 40.84
slip rate
31 mm/yr (25–36, 95% CI)
locking depth
22 km (15–30, 95% CI)
Loc: 3
30.50 40.66
slip rate
28 mm/yr (21–33, 95% CI)
locking depth
13 km (5–23, 95% CI)
Loc: 8
33.00 40.94
slip rate
27 mm/yr (21–34, 95% CI)
locking depth
15 km (8–24, 95% CI)
Loc: 4
31.00 40.58
slip rate
24 mm/yr (18–30, 95% CI)
locking depth
15 km (5–25, 95% CI)
Loc: 9
33.50 40.99
slip rate
26 mm/yr (21–32, 95% CI)
locking depth
16 km (8–25, 95% CI)
Loc: 5
31.50 40.67
slip rate
24 mm/yr (17–31, 95% CI)
locking depth
17 km (9–27, 95% CI)
Loc: 10
34.00 41.09

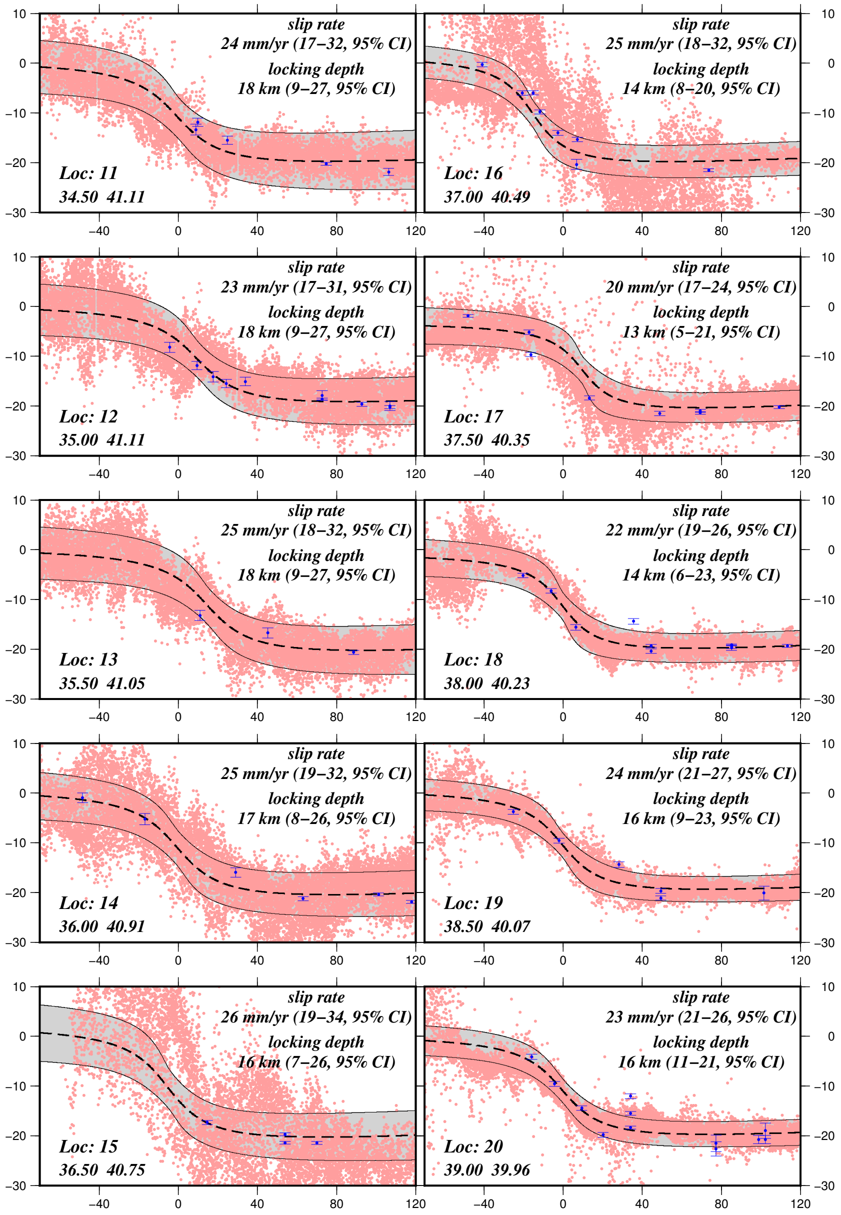

slip rate
24 mm/yr (17–32, 95% CI)
locking depth
18 km (9–27, 95% CI)
Loc: 11
34.50 41.11
slip rate
25 mm/yr (18–32, 95% CI)
locking depth
14 km (8–20, 95% CI)
Loc: 16
37.00 40.49
slip rate
23 mm/yr (17–31, 95% CI)
locking depth
18 km (9–27, 95% CI)
Loc: 12
35.00 41.11
slip rate
20 mm/yr (17–24, 95% CI)
locking depth
13 km (5–21, 95% CI)
Loc: 17
37.50 40.35
slip rate
25 mm/yr (18–32, 95% CI)
locking depth
18 km (9–27, 95% CI)
Loc: 13
35.50 41.05
slip rate
22 mm/yr (19–26, 95% CI)
locking depth
14 km (6–23, 95% CI)
Loc: 18
38.00 40.23
slip rate
25 mm/yr (19–32, 95% CI)
locking depth
17 km (8–26, 95% CI)
Loc: 14
36.00 40.91
slip rate
24 mm/yr (21–27, 95% CI)
locking depth
16 km (9–23, 95% CI)
Loc: 19
38.50 40.07
slip rate
26 mm/yr (19–34, 95% CI)
locking depth
16 km (7–26, 95% CI)
Loc: 15
36.50 40.75
slip rate
23 mm/yr (21–26, 95% CI)
locking depth
16 km (11–21, 95% CI)
Loc: 20
39.00 39.96

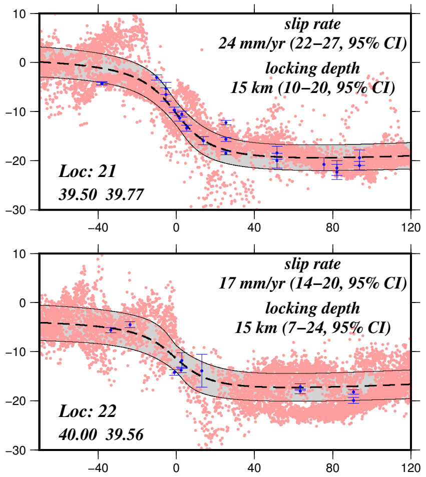

Supplementary Figure 6: Fault parallel velocity profiles with our MAP model solution. x-axis is distance from fault (km) and y-axis the fault parallel velocity (mm yr^{-1}). Locations are the indicated by open circles in Figure 3a in the paper with Loc: 1 corresponding to the western most point.

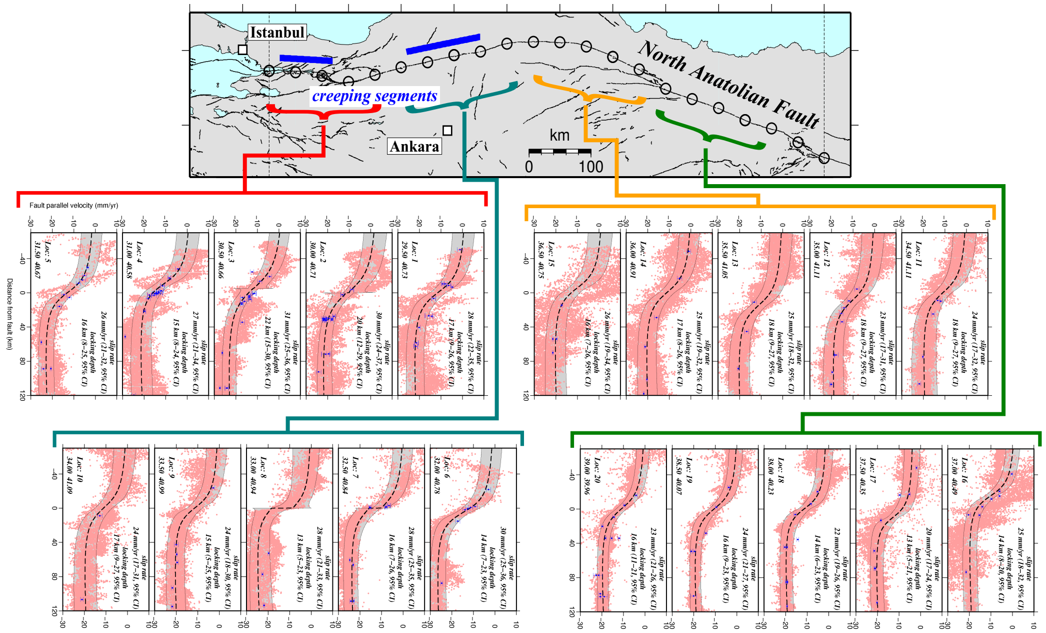

Supplementary Figure 7: Compilation image showing the location of each profile along the NAF.

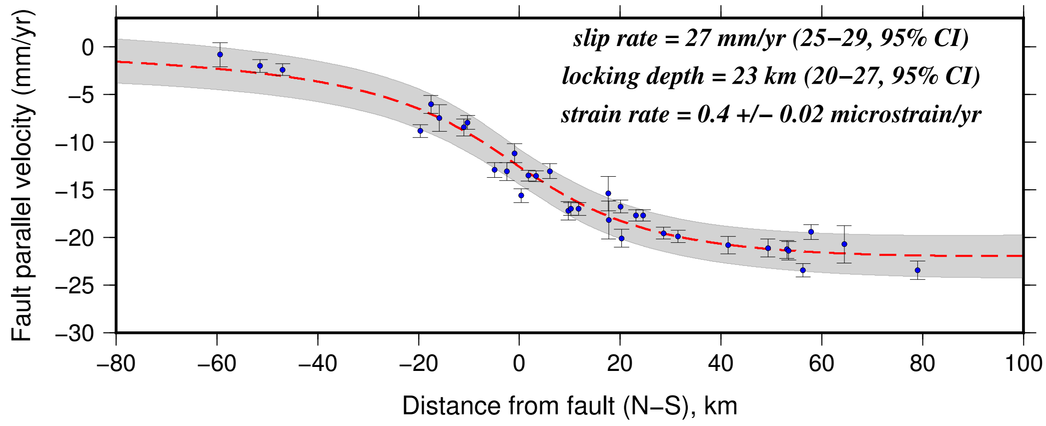

Supplementary Figure 8: Slip rate, locking depth and strain rate calculated for GNSS velocities over the Izmit region before the 1999 earthquake. Error bars are 1-sigma.

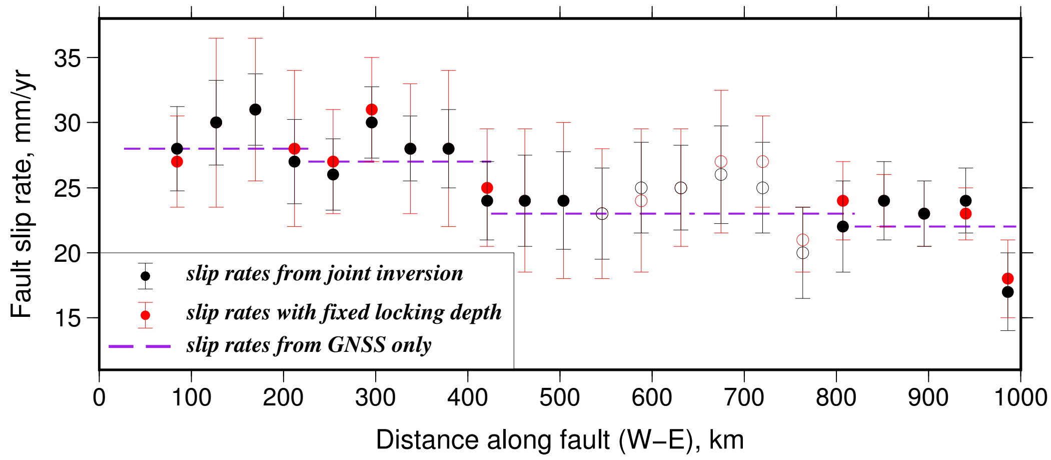

Supplementary Figure 9: Inversion results solving for both slip rate and locking depth compared with results with a fixed locking depth at 16 km and solving for just the slip rate. The solid circles are results from profiles that are in the region with both ascending and descending information (see polygons in Fig. 1c), while the open circles are profiles that are in areas where only ascending or descending data are available. The error bars represent the 68% confidence bound.

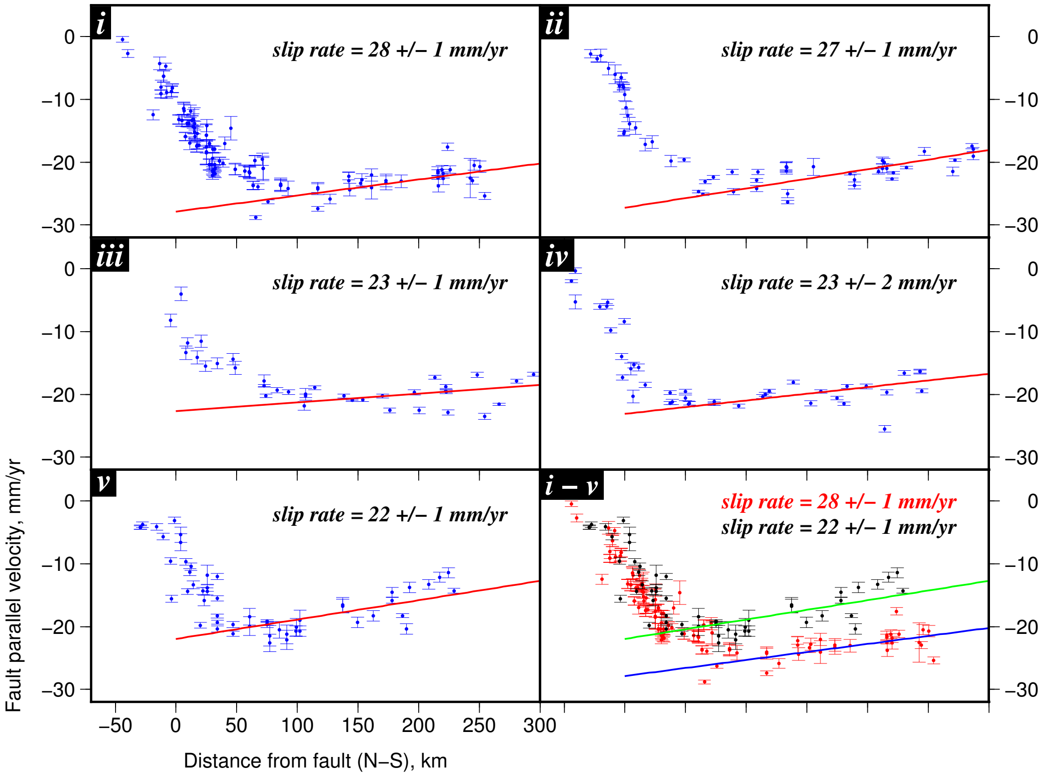

Supplementary Figure 10: Estimated slip rates from GNSS velocities in broad, ∼150 km wide profiles. The GNSS velocities are in a Eurasia-fixed reference frame and plotted with 1-sigma error bars. If we assume no internal deformation within central Turkey then the projection of far field GNSS velocities onto the fault gives the estimated slip rate from GNSS. The variation along the fault is mostly due to the anticlockwise rotation of Anatolia with respect to Eurasia. The last panel shows the velocities for profiles *i* and *v*, and clearly shows the difference in slip rate between the eastern and western regions due to the internal rotation of Anatolia.
